# Семейный случай синдрома фон Хиппеля-Линдау

**DOI:** 10.14341/probl13510

**Published:** 2025-12-02

**Authors:** Р. А. Атанесян, Л. Я. Климов, Т. М. Вдовина, Г. А. Санеева, Е. И. Андреева, И. К. Гаспарян

**Affiliations:** Ставропольский государственный медицинский университет; Краевой эндокринологический диспансерРоссия; Stavropol State Medical University; Regional endocrinological dispensaryRussian Federation; Ставропольский государственный медицинский университетРоссия; Stavropol State Medical UniversityRussian Federation; Краевой клинический перинатальный центр №1Россия; Stavropol Regional Clinical Perinatal Center №1Russian Federation

**Keywords:** синдром фон Хиппеля-Линдау, феохромоцитома, артериальная гипертензия, генетическое исследование, von Hippel-Lindau syndrome, pheochromocytoma, hypertension, genetic research

## Abstract

Синдром фон Хиппеля-Линдау (ФХЛ) — редкое аутосомно-доминантное заболевание, которое приводит к формированию синдрома множественной неоплазии. Патология в первую очередь вызвана инактивацией гена VHL, который расположен на хромосоме 3 (3p25/26) и кодирует фермент убиквитинлигазу, влияющую на гипоксией индуцируемый фактор-1α (HIF-1α). Генетический дефект сопровождается накоплением белка HIF-1α, активируя ключевые канцерогенные пути, а активированные цитокины вызывают избыточную пролиферацию опухолевых клеток и онкогенез. К настоящему времени зарегистрировано более 500 мутаций в VHL. Синдром ФХЛ характеризуется различными опухолями, включая гемангиобластомы сетчатки и центральной нервной системы, феохромоцитомы, кистозную аденому и другие.

В представленном клиническом описании феохромоцитома вначале была диагностирована у мамы пациента, а через 2 месяца у старшего сына. В последующем результаты молекулярно-генетического исследования позволили верифицировать диагноз, так как в гене в 3 экзоне VHL обнаружена замена одного нуклеотида в гетерозиготном состоянии с.500 G>А, приводящая к замене аминокислоты p.R167Q. Идентификация мутации гена VHL требовала генетического консультирования всех членов семьи, в ходе которого аналогичная мутация идентифицирована и у младшего брата. Хирургическое лечение, безусловно, является основным методом лечения синдрома ФХЛ, однако достижения в области генетической диагностики открывают новые горизонты в терапии данных пациентов.

## Abstract (en)

Von Hippel-Lindau syndrome (FHL) is a rare autosomal dominant disease that leads to the formation of multiple organ tumor syndrome. The pathology is primarily caused by the inactivation of the VHL gene, which is located on chromosome 3 (3p25/26) and encodes ubiquitin ligase, which destroys hypoxia-induced factor-1α (HIF-1α). The genetic defect leads to the accumulation of HIF-1a protein, activating key carcinogenic pathways, and activated cytokines cause abnormal proliferation of tumor cells and oncogenesis. To date, more than 500 mutations have been registered in VHL. FHL syndrome is characterized by various tumors, including hemangioblastomas of the retina and central nervous system, pheochromocytomas, clear cell renal cell carcinoma, cystic adenoma and others. In the presented clinical description, pheochromocytoma was initially diagnosed in the patient’s mother, and 2 months later in the eldest son. Subsequently, the results of a molecular genetic study made it possible to verify the diagnosis, since in the gene in exon 3 of VHL, a single nucleotide was replaced in the heterozygous state of C.500 G>A, leading to the replacement of the amino acid p.R167Q. Identification of the VHL gene mutation required genetic counseling of all family members, during which a similar mutation was identified in the younger brother. Surgical treatment is the main method of treating FHL syndrome, but advances in genetic research technologies provide new opportunities for the treatment of tumors associated with this syndrome.

## АКТУАЛЬНОСТЬ

Синдром фон Хиппеля-Линдау (ФХЛ) (цереброретинальный ангиоматоз, Von Hippel–Lindau disease) — редкое заболевание, наследуемое по аутосомно-доминантному типу с высокой пенетрантностью, сопровождающееся ростом опухолей в различных органах. При этом синдром ФХЛ представляет собой один из наиболее распространенных онкоассоциированных синдромов с частотой 1 случай на 36 000 новорожденных [1][10][18]. Мутация в 3-й (3p25/26) хромосоме, которая кодирует ген супрессора роста опухоли VHL, является причиной данного заболевания [2]. Ген VHL был идентифицирован в 1993 г., и к настоящему времени известно более 500 мутаций данного гена [2][3][35]. Белок VHL (pVHL), являющийся главным регулятором гипоксией индуцируемого фактора-α (HIF-α), кодируемый геном VHL, представлен 3 экзонами и состоит из двух доменов. Домен α в основном образует комплекс с элонгином B и элонгином C (VCB-CUL2), дополнительно составляя убиквитиназу E3, а домен β взаимодействует с HIF-α [4][5]. В физиологических условиях комплекс VCB-CUL2 напрямую воздействует на HIF-α, вызывая его деградацию, путем полиубиквитинирования (посттрансляционное присоединение ферментами убиквитинлигазами одного или нескольких мономеров убиквитина). Мутации в гене VHL могут снижать способность убиквитиназы E3 к деградации, что приводит к накоплению белка HIF-1α, который, в свою очередь, запускает ключевые канцерогенные пути, такие как ангиогенез, гликолиз, транспорт глюкозы и эритропоэз [6][7]. Активированные в процессе цитокины, такие как фактор роста эндотелия сосудов (VEGF), тромбоцитарный фактор роста (PDGF), эритропоэтин и трансформирующий фактор роста альфа (TGF-α) [8][9]][могут приводить к повышенной пролиферации опухолевых клеток, подавляя апоптоз и способствуя формированию опухоли [10].

В основе клинической классификации заболевания лежит корреляция генотип-фенотип [3][4].

Так, тип 1 синдрома ФХЛ характеризуется полной клинической картиной, но без феохромоцитомы (Ф) [11][12][13]. Напротив, тип 2 включает наличие Ф (риск 40–60%) и имеет несколько подтипов: подтип 2A — без рака почки (редко), подтип 2 B — с раком почки (часто) и подтип 2C: проявляется только Ф без других проявлений синдрома [11][14].

Спектр патогенных генетических вариаций при синдроме ФХЛ разнообразен, а ассоциации между генотипами и фенотипами не всегда предсказуемы [15]. Базы данных COSMIC содержат более 1800 записей генетических вариаций в гене VHL [16]. Наиболее распространенные генетические изменения, связанные с заболеванием ФХЛ, включают делеции экзонов, миссенс-мутации, вставки и делеции в рамке считывания, мутации сайта сплайсинга, вставки и делеции со сдвигом рамки считывания, а также структурные вариации и даже слияния генов [17][18]. При типе 2, независимо от подтипа, преимущественно обнаруживаются миссенс-мутации, которые, как было обнаружено, обычно сохраняют частичную активность белка VHL [18][19]. Важно понимать, что тип генетического варианта ФХЛ зависит от возраста [20]. Так, пенетрантность заболевания к 65 годам составляет более 90%, при этом у пациентов в возрасте 15 лет в 16% случаев было бессимптомное течение, а у 11% встречались симптоматические проявления синдрома [19][36]. Также было указано, что связанные с ФХЛ опухоли были зарегистрированы уже в возрасте 2 лет [19].

В детском возрасте болезнь ФХЛ отличается появлением неврологической симптоматики на фоне уже существующих зрительных нарушений. В некоторых случаях заболевание у детей манифестирует субарахноидальным кровоизлиянием, а на ранних стадиях синдром можно заподозрить лишь при офтальмоскопии, визуализируя гемангиобластому сетчатки [11]. Ф в педиатрической практике как изолированная опухоль встречается редко, и в случае ее диагностики должен инициироваться поиск наследственного синдрома. Ф в 35% случаев встречаются в рамках ФХЛ, в 25% случаев — при синдроме множественной эндокринной неоплазии 2 типа (МЭН) и в 14% — при нейрофиброматозе 1 типа [1][2][14]. Также необходимо отметить, что Ф как причина симптоматической артериальной гипертензии (АГ) встречается лишь у 0,5–2% детей [2]. Данная нейроэндокринная опухоль из хромаффинных клеток в большинстве случаев является доброкачественной, и только 10% имеют метастатический потенциал [1][5]. Среди пациентов с метастатическими Ф большинство случаев являются спорадическими, у пациентов с наследственными Ф чаще всего диагностируются мутации SDHB, что составляет до 43% случаев, за ними следуют мутации VHL, SDHD и NF1 [2][3]. Злокачественные Ф (критерии злокачественности включают проникновение в соседние органы, большую опухоль, наличие лимфаденопатии при визуализации или фиксацию при сцинтиграфии) редко встречаются при синдроме ФХЛ [21]. К сожалению, в настоящее время нет доказанных эффективных маркеров или методов для оценки риска рецидива или выявления злокачественности, однако ряд исследований показал, что низкая экспрессия факторов HIF1-α характерна для опухолей с более низким риском злокачественности [22][23].

Ф как часть синдрома ФХЛ имеет исключительно норадреналиновый фенотип. Биохимические маркеры опухоли могут помочь отличить VHL-ассоциированные Ф от Ф при синдроме МЭН 2-го типа. У пациентов с синдромом ФХЛ отмечена низкая экспрессия PNMT (фенилэтаноламин-N-метилтрансферазы), преобразующей норадреналин в адреналин [24][25]. Золотым стандартом в диагностике Ф является определение метанефрина и норметанефрина в плазме или в суточной моче, чувствительность данного метода составляет 99%, а специфичность — 85–89% [3][11][14].

Основными причинами смерти при синдроме ФХЛ являются гемангиобластома ЦНС (41–60%) и карцинома почек (27–47%). Однако для детей, у которых оба родителя имеют мутированный ген VHL, летальность при гемангиобластоме ЦНС увеличивается до 75,8%, а при карциноме почек наоборот составляет 15,7% [26].

Лечение синдрома ФХЛ в основном является хирургическим, устранение поражений в различных органах и системах, и, конечно, оно не может кардинально улучшить прогноз пациентов [27][28][29]. Следует рассмотреть возможность проведения органосохраняющей операции, которая считается успешной стратегией лечения. Исследования показывают низкий уровень рецидивов, отсутствие метастазирования и редкую зависимость от заместительной терапии после кортикальносберегающих методов [28][29]. Более того, операция по сохранению надпочечников не влияет на выживаемость. Операция по сохранению надпочечников является терапией выбора у детей.

С появлением технологии секвенирования и персонифицированной медицины диагностика и лечение синдрома ФХЛ открыли новую главу. Использование препаратов, блокирующих проангиогенез, пути передачи сигнала VEGF, может в определенной степени блокировать возникновение и развитие заболевания. Исследования показали, что использование анти-VEGF (препарата бевацизумаба) у пациентов с метастатическим раком почки может улучшить выживаемость пациентов [30]. Использование ингибитора рецепторной тирозинкиназы (сунитиниба) может уменьшать объем некоторых опухолей [31]. В 2018 г. открыта новая мишень VHL ZHX2 (цинковые пальцы и гомеобоксы 2), которая может регулировать сигнализацию ядерного фактора каппаB (NF-KB) в тканях с дефицитом VHL, способствуя опухолеобразованию, потенциально становясь новой мишенью для лечения [32]. В 2021 г. FDA США одобрило ингибитор HIF-2α (белзутифан) для лечения некоторых опухолей, связанных с синдромом ФХЛ, которые не требуют безотлагательного хирургического вмешательства [33]. С одобрением белзутифана развивающаяся технология секвенирования следующего поколения может дать толчок прецизионной медицине опухолей, связанных с синдромом ФХЛ.

## ОПИСАНИЕ СЛУЧАЯ

В настоящей статье описан случай семейной диагностики Ф у мальчика 11 лет и его мамы в рамках синдрома ФХЛ.

На приеме у детского эндокринолога мальчик А. 11 лет 9 месяцев с жалобами на слабость, тошноту, периодически — рвоту, эпизоды повышения АД до 160/80 мм рт.ст., наличие диагностированного образования в левом надпочечнике.

Анамнез заболевания: со слов пациента, около трех недель до обращения у него отмечалось повышение температуры тела до 40 °C, одновременно появилась боль в левом подреберье. Мальчик консультирован педиатром по месту жительства, был направлен на УЗИ почек. В ходе исследования диагностирован пиелонефрит, назначена антибактериальная терапия. Проводимая терапия была неэффективна, ввиду чего было рекомендовано повторно выполнить УЗИ почек. В ходе повторного УЗИ мочевыделительной системы в левом надпочечнике диагностировано образование (размерами 5,0*5,0*5,5 см, V=74 см³). Пациент был направлен на госпитализацию в НМИЦДОГ им. Н.Н. Блохина (к тому времени мама пациента находилась в этом же центре). В процессе активного опроса выяснилось, что около 5 месяцев назад у мальчика в школе отмечалась головная боль, повышение АД до 160/80 мм рт.ст. Мальчик консультирован по месту жительства, выставлен диагноз «ВСД пубертатного периода».

Анамнез жизни: ребенок от 1-й беременности, протекавшей на фоне уреаплазмоза, гестоза в I половине беременности, ОРВИ в сроке 28 недель, 1-х срочных родов в возрасте 19 лет. Вес при рождении — 2680 г, длина — 49 см. В неонатальном периоде — длительная неонатальная желтуха. Из родильного дома выписан на 6-е сутки в удовлетворительном состоянии. На грудном вскармливании — до 1 года 7 месяцев. Родители кровными родственниками не являются.

Генеалогический анамнез: известно, что у мамы во время второй беременности с 28-й недели гестации диагностирована АГ, принимала гипотензивные препараты. Во время третьей беременности АГ отмечалась с 10-й недели гестации, по поводу чего пациентке было назначено обследование, в том числе консультация офтальмолога, диагностирована гипертоническая ангиопатия. По рекомендации врача-офтальмолога выполнено УЗИ почек, диагностированы образования в надпочечниках (2 месяцами ранее до обращения старшего сына), беременность прервана по медицинским показаниям. У бабушки по линии матери — внутричерепная опухоль.

На момент обращения ребенка мама пациента находилась в стационаре НМИЦДОГ, где была выполнена резекция правого надпочечника и адреналэктомия слева, назначен гидрокортизон 20 мг/сутки (заместительная гормональная терапия).

Объективный осмотр: рост — 159 см (SDS L=+0,78), вес — 50 кг, ИМТ — 19,8 кг/м² (SDS BMI=+0,78). Физическое развитие соответствует возрасту. Общее состояние удовлетворительное. Распределение подкожно-жировой клетчатки равномерное. Кожные покровы смуглой окраски, умеренно влажные, кофейные пятна по телу в количестве 4. Фенотип без особенностей. Дыхание везикулярное, хрипов при аускультации нет. Тоны сердца звучные, ритмичные. АД — 103/63 мм рт.ст. (измерение выполнено 3-кратно на правой руке после 5 минут отдыха). Щитовидная железа не увеличена, эластичной консистенции, безболезненная, подвижная. Наружные половые органы развиты по мужскому типу, 3 стадия по Таннеру (G3P3).

## РЕЗУЛЬТАТЫ ФИЗИКАЛЬНОГО, ЛАБОРАТОРНОГО И ИНСТРУМЕНТАЛЬНОГО ИССЛЕДОВАНИЙ

В НМИЦДОГ им. Н.Н. Блохина пациенту выполнено лабораторно-инструментальное обследование: гормональное обследование (кортизол крови утром — 16,4 мкг/дл (норма), норметанефрины в суточной моче — 536 мкг/сут (55,0–277,0 мкг/сут), метанефрины в суточной моче в норме. Выполнено УЗИ органов брюшной полости, забрюшинного пространства — забрюшинно паравертебрально слева описано объемное образование овальной формы, с четкими, ровными контурами, размерами 65х47х52 мм, структура образования неоднородная, солидная, с мелкими кистозными включениями. Результаты КТ органов грудной клетки: в S2 справа — субплеврально уплотнение (спайка). Очаговые и инфильтративные изменения в легких не выявлены. Внутригрудные лимфоузлы не увеличены. МР-исследование брюшной полости — забрюшинно, паравертебрально слева на уровне Th12, L1 позвонков определяется объемное образование округлой формы размерами 5,0х4,9х5,5 см, с четкими ровными контурами. Структура образования неоднородная — солидная по периферии с интенсивным контрастным усилением на постконтрастных сканах. Центральные отделы выполняет неоднородный субстрат с гиперинтенсивными включениями на Т1 ИП, накапливающий контрастный препарат. Опухоль несколько компримирует верхний полюс левой почки по передней поверхности.

Пациентам с подтвержденной Ф до оперативного лечения необходимо проводить ПЭТ или сцинтиграфию с 123I-метайодбензилгуанидином (MIBG) с целью выявления вненадпочечниковой локализации или метастазов. Высокая специфичность отмечена при применении сцинтиграфии с MIBG, который накапливается в клетках хромаффинной ткани и включается в процесс синтеза катехоламинов. Результаты сцинтиграфии с MIBG пациента: на сцинтиграммах всего тела определяется очаг повышенного патологического накопления радиофармацевтического препарата (РФП) в области опухолевого образования левого надпочечника. Распределение РФП в структуре опухоли носит гетерогенный характер с частично негативным ядром, основная активность сосредоточена в периферических отделах — ОН ср.=267%. Картина соответствует феохромоцитоме левого надпочечника без явных признаков диссеминации.

КТ органов брюшной полости с внутривенным контрастированием: забрюшинно слева определяется объемное образование округлой формы, неоднородной солидно-кистозной структуры, размерами 4,9х4,8х5,4 см. При внутривенном контрастировании отмечается активное накопление контрастного вещества в периферических отделах опухоли. Образование прилежит к телу и латеральной ножке левого надпочечника, без четкой границы с ними, к передней поверхности левой почки, оттесняя и компримируя, распространяясь до уровня ворот, прилегая к почечным артериям и вене, без четких признаков вовлечения в процесс. Поджелудочная железа оттеснена краниально. Печень, селезенка, поджелудочная железа — без очаговых изменений. ЧЛС не расширены. Парааортально определяется единичный лимфатический узел 0,9х0,7 см. Свободной жидкости в брюшной полости не выявлено. Образование левого надпочечника (феохромоцитома?). Пациенту в рамках предоперационной подготовки назначен прием доксазозина. Взят анализ крови для исследования ДНК панели генов, ассоциируемых с онкогенетическими синдромами.

В совокупности данных генеалогического анамнеза, данных объективного осмотра и наличия подтвержденной Ф (в послеоперационном периоде) были предположены следующие диагнозы: синдром ФХЛ, нейрофиброматоз 1 типа и синдром МЭН 2 типа. Характеристика дифференциальной диагностики заболеваний, сопровождающихся наличием Ф, представлен в таблице 1.

**Table:** Таблица 1. Дифференциальная диагностика заболеваний, протекающих с повышенным риском феохромоцитомы Примечание. НФ1 — нейрофиброматоз 1 типа; MЭН2A — множественная эндокринная неоплазия 2А типа (МЭН2А); МРЩЖ — медуллярный рак щитовидной железы; MЭН2B — множественная эндокринная неоплазия 2В типа (МЭН2В).

Ген	Заболевание/ синдром	Клиническая характеристика
МАКС SDHA SDHAF2 SDHB SDHC SDHD TMEM127	Наследственный параганглиома-феохромоцитомный синдром	Характеризуется феохромоцитомами и параганглиомами
NF1	Нейрофиброматоз 1	Пятна цвета кофе с молоком, интертригинозные веснушки, кожные нейрофибромы и неспособность к обучению. Феохромоцитомы чаще встречаются у лиц с НФ1, но все еще встречается редко и встречается у <1% взрослых с НФ1
РЕТ	Множественная эндокринная неоплазия 2 типа (МЭН2)	MЭН 2A: риск медуллярного рака щитовидной железы (МРЩЖ), феохромоцитомы и аденомы/гиперплазии паращитовидных желез. Феохромоцитомы обычно появляются после МРЩЖ или одновременно; однако они являются первым проявлением в 13–27%. MЭН 2B: невриномы слизистой оболочки губ и языка, ганглионевроматоз желудочно-кишечного тракта, марфаноподобная внешность и повышенный риск МРЩЖ и феохромоцитомы. Феохромоцитомы встречаются у 50% людей с MЭН 2B

Пациенту выполнено хирургическое лечение: срединная лапаротомия, адреналэктомия слева, дренирование брюшной полости. При ревизии брюшной полости диссеминации по печени и брюшине не выявлено. Получены результаты микроскопического описания послеоперационного материала: феохромоцитома надпочечника. Определяется остаточная ткань надпочечника. Полиморфизм ядер клеток (по Fuhrman)-2. Митозы: 4 mf/50 HPF. Очаги некроза 35% ткани опухоли. Признаки ангиолимфатической инвазии не обнаружены. Сформирована фиброзная капсула узла опухоли. Транскапсулярная инвазия феохромоцитомы в прилежащую жировую ткань не обнаружена. Регионарные лимфоузлы не обнаружены.

Через 2 месяца после оперативного лечения пациент явился на плановый прием к эндокринологу по месту жительства. Ребенок заместительную гормональную терапию гидрокортизоном не получал. На приеме у эндокринолога предоставлены результаты обследования: электролиты крови (натрий — 140 ммоль/л (норма), калий — 5,1 ммоль/л (норма), ионизированный кальций — 1,41 (1,12–1,32), норметанефрин —133,20 мкг/сут (55,0–277,0 мкг/сут), метанефрин — 94,20 мкг/сут (59,0–188,0 мкг/сут). Выполнен биохимический анализ крови (глюкоза крови, магний, фосфор) — без отклонений.

Назначено расширенное гормональное обследование: ТТГ, СТ4, АТ-ТПО, АТ-ТГ — без отклонений, паратиреоидный гормон (ПТГ) — 48,85 пг/мл (2,5–25), кальцитонин — 1,4 пг/мл, ЛГ, ФСГ, тестостерон, пролактин, кортизол, ИФР — 312 нг/мл (норма) — без отклонений.

Выполнено УЗИ щитовидной железы — признаки умеренно выраженных диффузных изменений паренхимы щитовидной железы. Очаговые изменения правой доли (узел в стадии формирования?). Фокальные изменения коллоидного характера. Категория EU THI-RADS II (Thyroid Imaging Reporting and Data System) [35].

В рамках скрининга сопутствующих новообразований выполнено МРТ головного мозга с контрастированием на серии МР-томограмм головного мозга, выполненных в последовательностях Т1, Т2, FLAIR в аксиальной, сагиттальной и коронарной плоскостях признаки умеренной структурной неоднородности аденогипофиза, данных за объемный процесс не выявлено.

Пациенту было рекомендовано повторно выполнить исследование ПТГ — 97,8 пг/мл (12–95). Возникли предположения, что гиперпаратиреоз носит вторичный характер, обусловлен гиповитаминозом D (суммарный уровень витамина D у пациента — 10,1 нг/мл — дефицит).

В ходе динамического наблюдения через 2 месяца на фоне дотации холекальциферолом отмечалась нормализация показателя кальция крови и ПТГ.

Через 4 месяца после оперативного лечения были получены результаты МГИ, в гене VHL, в 3 экзоне обнаружена замена одного нуклеотида в гетерозиготном состоянии с.500 G>А, приводящая к замене аминокислоты p.R167Q, описанный вариант очень редко встречается в базах данных аллельных вариантов человека и в научной медицинской литературе описан как патогенный (МГИ выполнялось на базе лаборатории НМИЦ при поддержке Фонда поддержки и развития филантропии «КАФ», проведено полное секвенирование экзома).

Хакер и др. определили, что мутации p.R167Q, расположенные в области связывания элонгина-C, вероятно, сохранили способность связывать комплексные белки VCB-CUL2 и элонгин-B, как показано в экспериментах по коиммунопреципитации [36]. Несмотря на нестабильную ассоциацию белков, составляющих комплексы VBC, это исследование показало, что мутация p.R167Q VHL может убиквитинировать HIF-1α, так Buart et al. показали, что мутация R167Q VHL приводит к молекулярным изменениям, связанным с более выраженной стволовостью раковых клеток и пластичностью опухоли [37].

Таким образом, результаты МГИ позволили идентифицировать специфическую мутацию гена VHL. Верификация синдрома ФХЛ требует генетического тестирования данной мутации у всех членов семьи. Младшему брату было выполнено секвенирование по Сенгеру, в результате которого также выявлен вариант с.500 G>А (pArg167Gln).

## ОБСУЖДЕНИЕ

Протокол скрининга на синдром ФХЛ у детей с положительными мутациями с клиническими проявлениями или без них рекомендует проводить поиск ангиомы сетчатки, начиная с раннего возраста, а именно выполнять офтальмологическое обследование 1 раз в год, также мониторинг АД, метаболиты катехоламинов и МРТ брюшной полости каждые 12 месяцев начиная с 8 лет, что важно для скрининга Ф [38].

Для детей с клиническими проявлениями синдрома ФХЛ УЗИ и/или МРТ брюшной полости каждые 12 месяцев с 16 лет необходимо для наблюдения за раком почек и опухолями поджелудочной железы, однако у детей без клинических проявлений наблюдение рекомендуется с 8 лет [38].

Очевидно, что пациенты с верифицированным диагнозом нуждаются в скрининге осложнений, тщательном наблюдении онколога с целью предотвращения серьезных осложнений.

Известно, что спустя 2 года после оперативного лечения у мамы пациента диагностирован рецидив Ф правого надпочечника, по поводу чего повторно выполнена резекция правого надпочечника.

## ЗАКЛЮЧЕНИЕ

Раннее и агрессивное лечение синдрома, несмотря на возможность возникновения множественных рецидивов, может улучшить прогноз и увеличить выживаемость пациентов. Поскольку синдром является аутосомно-доминантным заболеванием, бремя болезни, несомненно, огромно. Существует мало доклинических и клинических исследований по этой теме, и симптоматическое лечение является основой, однако есть надежда, что в ближайшем будущем препараты или методы лечения, нацеленные на генетические мишени, будут подавлять экспрессию мутированных генов еще до рождения детей с высоким риском [39]. Генетическое исследование членов семьи пациента с установленным диагнозом должно носить обязательный характер [40].

Таким образом, проблема диагностики синдрома ФХЛ представляется мультидисциплинарной и требует квалифицированной оценки биохимических, нейрогормональных, радиологических и генетических методов исследований. Оперативное лечение, таргетная лекарственная и генная терапия позволят пациентам с синдромом ФХЛ получить более широкие и эффективные возможности терапии. Залогом успешного лечения пациентов с синдромом ФХЛ очевидно является своевременный скрининг ассоциированной патологии.

## ДОПОЛНИТЕЛЬНАЯ ИНФОРМАЦИЯ

Источники финансирования. Работа выполнена по инициативе авторов без привлечения финансирования».

Конфликт интересов. Авторы декларируют отсутствие явных и потенциальных конфликтов интересов, связанных с содержанием настоящей статьи. Материал данной статьи частично был опубликован в сборнике тезисов III Конференции по орфанным и детским эндокринным заболеваниям «Молекулярно-генетические исследования в практике детского эндокринолога», 28–29 марта 2023 г.

Участие авторов. Все авторы одобрили финальную версию статьи перед публикацией, выразили согласие нести ответственность за все аспекты работы, подразумевающую надлежащее изучение и решение вопросов, связанных с точностью или добросовестностью любой части работы.

Согласие пациента. Законный представитель пациента (мама) добровольно подписал информированное согласие на публикацию персональной медицинской информации в обезличенной форме в журнал «Проблемы эндокринологии».

Благодарности. Выражаем благодарность сотрудникам НМИЦДОГ им. Н.Н. Блохина, также сотрудникам ГНЦ РФ ФГБУ, программе «Альфа-эндо» за возможность проведения молекулярно-генетического исследования пациентам с эндокринной патологией.
